# Post-traumatic stress disorder in an antenatal population in South Africa: Prevalence and associated factors

**DOI:** 10.4102/sajpsychiatry.v31i0.2504

**Published:** 2025-12-20

**Authors:** Megan Fyffe, Mojalefa Makgata, Lesley Robertson

**Affiliations:** 1 Department of Psychiatry, Faculty of Health Sciences, University of the Witwatersrand, Johannesburg, South Africa; 2 Department of Health and Mental Health, University of the Witwatersrand, Sedibeng, South Africa; 3 Department of Psychology, Faculty of Health Sciences, University of the Witwatersrand, Johannesburg, South Africa

**Keywords:** antenatal, community health centre, prevalence, posttraumatic stress disorder, PCL-5

## Abstract

**Background:**

The prevalence of post-traumatic stress disorder (PTSD) is uncertain globally, and there is a paucity of information in South Africa. Perinatal PTSD may impair maternal functioning and negatively affect their parenting ability.

**Aim:**

This study aimed to determine the prevalence and associated factors of PTSD in an antenatal population in South Africa.

**Setting:**

The study was conducted at Johan Heyns Community Health Centre in Sedibeng district, Gauteng province.

**Methods:**

A cross-sectional questionnaire-based survey was conducted in which the Post-traumatic Stress Disorder Checklist for DSM-5 (PCL-5) was administered among women attending the Johan Heyns antenatal clinic. The PCL-5 is a validated screening tool for PTSD, which comprises 20 items scored on a 5-point Likert scale for symptom severity. A total score ≥ 31 indicates at least moderate PTSD.

**Results:**

Of the 98 pregnant women who participated in the study, 53 (54%) reported having experienced or witnessed a traumatic event. Total PCL-50 scores ranged from 0–65 (mean 13.8 standard deviation [s.d. = 18.1]), with at least one intrusion symptom endorsed by 37 women, followed by cognition and mood change by 36, arousal and reactivity by 32, and avoidance by 24. Sixteen women (16.3%) screened positive for PTSD (mean score 49.2 [s.d. = 12.7]). While univariate analysis found no significant associations with PTSD, alcohol use was associated with an increased risk of PTSD on multivariate analysis (RR 2.7; [95% confidence interval [CI] 1.1–7.0; *p* = 0.036).

**Conclusion:**

Post-traumatic stress disorder is common among antenatal women in Sedibeng district and may be associated with alcohol use in pregnancy.

**Contribution:**

This study highlights the importance of screening pregnant women for PTSD to ensure early intervention and management.

## Introduction

Post-traumatic stress disorder (PTSD) results in impairment in functioning in several important areas such as social, interpersonal, vocational and academic functioning.^1^ According to the World Health Organization (WHO), about 70% of people may experience a traumatic event during their lives globally, of whom 5.6% develop PTSD, with rates varying depending on the type of trauma.^2^ Among the South African general population, the South African Stress and Health (SASH) study found lifetime and 12-month prevalence rates of PTSD of 2.3% and 0.6%, respectively.^3^ A secondary analysis of the SASH study found lifetime and 12-month prevalence rates of 3.5% and 0.7%, respectively, among those who had experienced at least one traumatic event in their lifetime (i.e. among 74% of SASH study respondents).^4^

Post-traumatic stress disorder tends to be more common among women than men, especially following sexual trauma.^2^ This is a concern in South Africa which has high rates of gender-based violence. A national survey by the Human Sciences Research Council found 35% of adult women have a lifetime experience of physical and/or sexual trauma.^5^ In the Gauteng province of South Africa, a household survey conducted among 500 women in 2010 found that 50% had experienced intimate partner violence (IPV) in their lifetime and 11.6% screened positive for PTSD.^6^

The impairment in functioning among women with PTSD has particular relevance for the perinatal period, when PTSD may have potentially devastating consequences with respect to negative effects on various child outcomes and mother–infant bonding and attachment.^7,8,9^ Several mechanisms for the associations between maternal PTSD, preterm delivery, low birth weight and poor infant development have been hypothesised. Stress within pregnancy has been linked to neuroendocrine alterations in cortisol, vasopressin and oxytocin as well as activation of the hypothalamic-pituitary adrenal axis in the baby.^10,11^ Furthermore, epigenetic modifications in children born to mothers with PTSD may additionally contribute to poorer infant neurodevelopment through glucocorticoid receptor methylation.^12^

A systematic review and meta-analysis of 35 studies examining PTSD during pregnancy by Yildiz et al.^13^ found prevalence rates ranging from 0% to 40% with a mean prevalence of 4.6%. Risk factors included hyperemesis gravida or a maternal history of childhood trauma. The prevalence of PTSD in studies that recruited women with high-risk pregnancies, medical and/or mental illness, or previous trauma was just under 19%, whereas it was 3.3% among women recruited from community-based antenatal services or maternity hospitals. Yildiz et al. found that, despite considerable amounts of research being carried out around PTSD, the understanding of PTSD and its associated factors within pregnancy is limited. In addition, very limited research has been conducted in low- and middle-income countries (LMICs).

The South African context provides unique opportunities for further research and work in the field of psychological trauma, exposure to trauma and its resultant effects on health, not limited to but including PTSD within the antenatal period. Intervention, management and treatment of individuals with severe PTSD requires intense, multifaceted biopsychosocial therapeutic interventions. Given the crucial role of maternal mental health in child development and attachment, early identification and treatment of antenatal PTSD is essential to improving outcomes for both mother and infant. This study aimed to determine the prevalence of PTSD symptoms among antenatal attendees at a district Community Health Centre (CHC) in Gauteng province and identify associated factors.

## Research methods and design

### Study design

A cross-sectional study design was conducted in which an investigator-administered questionnaire was used among pregnant women attending an antenatal clinic in the Sedibeng district of Gauteng province.

### Setting

The antenatal clinic was at Johan Heyns Community Health Centre (CHC), which is in an industrial city of the Sedibeng district in Gauteng province and serves a mixed suburban, informal settlement and rural population. This CHC was chosen because it has a large and well-established antenatal clinic as well as a mental health care service with doctors, nurses, social workers, and psychologists available to aid patients needing escalation of care. The CHC has facilities for uncomplicated deliveries. Women identified at the antenatal clinic as having high-risk pregnancies are referred to the regional hospital for further antenatal and obstetric care. As part of routine antenatal care, women attending the antenatal clinic are screened for mental health issues by a professional nurse using a standard set of screening questions in the government-issued maternity case record. A 3-item tool validated in South Africa has been implemented as standard antenatal screening for perinatal common mental illnesses and suicidality.^14^ This tool screens for symptoms of anxiety, depression and self-harm thoughts over a 2-week period. Women answering yes to two or more questions are referred to a registered counsellor as per the tool recommendations.

### Study population and sampling strategy

The study population comprised pregnant women following up at Johan Heyns antenatal clinic. To be enrolled in the study, participants needed to be 18 years of age or older and have a basic proficiency in English, as all questionnaires were administered in English. Participants were recruited through convenience sampling on the day of their antenatal clinic follow-up. Convenience sampling was chosen for its simplicity, cost-effectiveness and practicality in a resource-limited setting. All women waiting for their antenatal appointment were informed of the study aims and objectives and invited to participate. Women willing to participate were taken to a private interview room in the clinic, consent was obtained, and study questions were administered. Exclusion criteria were women who were too physically unwell to participate and women without capacity to consent to the traumatic questions being asked in the questionnaire due to health concerns at the time of interview.

Calculation of sample size was conducted using Glenn Israel tables.^15^ The tables provide sample sizes needed for a confidence interval (CI) of 95% and a population variability of 0.5 (i.e. the maximum degree of variability is assumed). A 10% leeway on precision was applied as this is the first study of its kind to be conducted in this setting. As Sedibeng district has an estimated population of 1 039 908 people from the 2022 census,^16^ 100 recorded responses would be necessary to obtain a CI of 95% with 10% precision.

### Study tools

#### Post-traumatic stress disorder checklist for DSM-5

The Post-traumatic Stress Disorder Checklist for DSM-5 (PCL-5) developed by the National Centre for PTSD at the US Department of Veterans Affairs^17^ was used to screen for and make a provisional diagnosis of PTSD. The PCL-5 consists of 20 items in five symptom clusters representing the DSM-5 criteria for PTSD, with Cluster B (intrusion) being items 1–5, Cluster C (avoidance) items 6–7, Cluster D (negative alterations in cognition and mood) items 8–14 and Cluster E (arousal and reactivity) items 15–20. Each item is rated according to how much the person is affected by the symptoms using a 5-point Likert scale with 0 for not at all and 4 for extremely.

A provisional diagnosis of PTSD may be determined from the PCL-5 in two ways: either through summing all 20 items from clusters B–E to arrive at a total score ranging between zero and 80 or by using DSM-5 diagnostic criteria. The National Centre for PTSD recommends using a total score with a cut-off score between 31 and 33 as psychometric studies indicate this to be more reliable for identifying at least moderate PTSD. The precise cut-point should be determined on a case-by-case basis and, in vulnerable populations, a lower cut-off score may apply to ensure no cases are missed, noting that the lower score may result in some false positives. As our study population is vulnerable, being pregnant women living in a society with a high level of interpersonal violence, we used the lower cut-off score of 31 to identify at least moderate PTSD in our study and to analyse associated factors.

When using DSM-5 criteria, an item is only endorsed as a symptom of PTSD if it is rated as 2 or more (i.e. experienced at least moderately). A diagnosis of PTSD requires endorsement of at least six items (one in each of clusters B and C and two in each of clusters D and E). To understand the nature of symptoms experienced by our study population, both methods were used to calculate PTSD probability.

#### Socio-demographic and clinical questionnaire

A questionnaire was developed by the principal investigator (Megan Fyffe) to obtain relevant socio-demographic and clinical variables, including age, population group, level of education, relationship status, employment, housing, antenatal, medical and substance use history. A population group was included to determine if the study sample is representative of the region and was documented using national census terminology.

### Data collection

Women were informed of the study, provided with an information leaflet, and invited to participate while in the antenatal waiting room by Megan Fyffe. Those who wanted to participate were taken to a private consultation room and given an opportunity to ask questions before signing the consent. Socio-demographic, antenatal, medical and substance use data were collected and the PCL-5 was administered by Megan Fyffe. Antenatal data were captured from the maternal health record and during the interview. Participants were monitored for any signs of distress during the interview process. All women who exhibited distress, screened positive for PTSD, or were using substances were referred to an on-site mental health professional. A deliberately low threshold for referral was maintained given the vulnerability of the study population and the possibility of complex biopsychosocial causes of the distress.

The interviews were conducted in English. Participants were assured of anonymity, with each interviewee assigned a study number and all data collected were stored separately from any identifying information. Participants could choose to skip questions on the socio-demographic questionnaire and remain in the study. An effort was made to ensure a supportive and safe environment for women choosing to participate.

### Data analysis

Data from all questionnaires were captured on a Microsoft Excel spreadsheet and imported into IBM SPSS version 29 for statistical analysis. Descriptive statistics including numbers, frequency distributions and means were used to summarise participants’ socio-demographic data and responses to PCL-5. Frequencies and percentages were presented for categorical variables as overall and stratified by PTSD symptoms (negative vs. positive).

Cronbach’s alpha was used to test for the reliability and consistency of the items of the PTSD scale. The tables stratified by PTSD symptoms were presented with denominators, referring to the number of participants who responded to a question and/or item. For continuous measures, means and standard deviations (s.d.) as well as medians and interquartile ranges were presented. A bar chart was used to report PTSD severity among participants with positive PTSD symptoms while a box and whisker plot provides graphical representational was used to report the means and s.d. for positive PTSD symptoms as well as the clusters of participants with positive PTSD symptoms.

To determine the statistical difference between participants with negative and positive PTSD symptoms, Chi-Square test or Fisher’s test was used where appropriate. For statistical comparison in continuous measures, medians were compared using a non-parametric test (Kruskal–Wallis test). Generalised linear model, using a log binomial distribution link function, was used to assess factors associated with positive PTSD symptoms. This model was selected because the proportion of participants with positive PTSD symptoms was > 10%. No variables were significant at univariate analysis; therefore, all variables in the univariate analysis were included in the multivariate model, and a backward selection procedure was used to reach the final model. Results for the model are presented as relative risks (RR), 95% CIs and *p*-values. Statistical significance was defined as *p*-value ≤ 0.05. Statistical analysis was conducted using SAS Enterprise Guide 7.1 (SAS Institute Inc., Cary, North Carolina, United Sates).

### Ethical considerations

An effort was made to ensure that consent to participate was voluntary. A distress protocol was put in place, and on-site access to mental health professionals was confirmed. All data were anonymised with the use of separately stored study numbers for each participant. Ethical consent for the study was approved by the Faculty of Health Sciences Human Research Ethics Committee of the University of the Witwatersrand (M240414MED24-03-477), and permission to conduct the study was provided by the Acting Chief Director of the Sedibeng District Health Services and the Operational Manager of Johan Heyns Clinic.

## Results

### Socio-demographic characteristics

The study sample comprised 98 women. Although 100 women initially participated in the study, one was excluded when it became apparent that she was under 18 years old. Another woman withdrew from the study as she experienced marked distress during the PCL-5 questionnaire, did not wish to continue and was referred to the mental health care services.

Socio-demographic characteristics are described in Table 1. The median age of participants was 26 years with 47 participants being between 25 and 34 years of age. Most participants were black African (84%), while white, mixed race and Indian and/or Asian population groups formed 10%, 5% and 1% of the sample, respectively. Just over a third (35%) of participants were single, and a high proportion (67%) had a monthly income of less than R5999. Only four participants reported earning over R10 000.00, while four preferred not to disclose their income status to the interviewer. A third (33%) of participants received a childcare support grant, while two participants received disability grants, both for bipolar disorder. Income insecurity was further underscored with 66 participants being unemployed.

**TABLE 1:** Demographic characteristics of participants.

Variable	*N*	%	Median	IQR	Mean	s.d.
**Age (in years)**	-	-	26.0	21.0–31	26.5	5.82
18–24	41	41.84	-	-	-	-
25–34	47	47.96	-	-	-	-
35–44	10	10.20	-	-	-	-
**Population group**	-	-	-	-	-	-
Black people	82	83.67	-	-	-	-
White people	10	10.20	-	-	-	-
Mixed race people	5	5.10	-	-	-	-
Asian people	1	1.02	-	-	-	-
**Relationship status**	-	-	-	-	-	-
Single	34	34.69	-	-	-	-
Living together	33	33.67	-	-	-	-
Married	30	30.61	-	-	-	-
Divorced	1	1.02	-	-	-	-
**Level of education**	-	-	-	-	-	-
Mainstream Grade 0–7	2	2.04	-	-	-	-
Mainstream Grade 8–11	14	14.29	-	-	-	-
Mainstream Grade 12	40	40.82	-	-	-	-
Special needs grade 10–12	5	5.10	-	-	-	-
Post high school	37	37.76	-	-	-	-
**Monthly income**	-	-	-	-	-	-
< R5999	63	67.02	-	-	-	-
R6000–R9999	27	28.72	-	-	-	-
≥ R10 000	4	4.26	-	-	-	-
**Housing type**	-	-	-	-	-	-
Informal	4	4.08	-	-	-	-
Rented room	45	45.92	-	-	-	-
Apartment/Flat	16	16.33	-	-	-	-
House	33	33.67	-	-	-	-
**Number of people in the household**	-	-	3	2–4	2.92	1.72
1	20	20.62	-	-	-	-
2–4	64	65.98	-	-	-	-
5–9	13	13.40	-	-	-	-
**Do you receive a disability grant?**	-	-	-	-	-	-
No	63	64.29	-	-	-	-
Yes	2	2.04	-	-	-	-
**Child care support grant**	33	33.67	-	-	-	-
**Currently employed?**	-	-	-	-	-	-
No	66	67.35	-	-	-	-
Yes	32	32.65	-	-	-	-

IQR, interquartile range; s.d., standard deviation.

With respect to living conditions, 46% of participants indicated they resided in rented rooms, and a large majority (66%) of participants lived in households with 2–5 people, while 20% lived alone. Educational attainment was limited, with only 40 participants having achieved matric and five having attended special education schools.

### Maternal health factors and substance use history

Maternal health factors and substance use history are outlined in Table 2. Half of the participants involved were primigravida and most (69%) were in their second or third trimester. Complications in previous pregnancies were reported by 16 participants. Eight participants reported having had previous contact with mental health services: five had received at least one counselling session (one after experiencing a stillbirth, one for suicidality and three for undisclosed reasons); and three had received psychiatric care (for PTSD, bipolar disorder and depression, respectively). The mental health screening tool in the maternity health record was completed in all participants, with one participant scoring 3, four scoring 2, one scoring 1 and 92 scoring 0. However, there was no documentation on whether counselling had been offered or a referral provided.

**TABLE 2:** Maternal health factors and substance use history.

Variable	*N*	%
**Gestational age (months)**		
Unsure	18	18.37
3	13	13.27
3–6	34	34.69
6–9	33	33.67
**Number of other children**		
None	49	50.00
One	30	30.61
More than one	19	19.39
**Prior pregnancy complications**		
No and/or NA	82	83.67
Yes	16	16.33
**Specify prior pregnancy complications**		
Antepartum haemorrhage	1	6.25
Caesarean section	2	12.50
Miscarriage	6	37.50
Preterm labour	3	18.75
Other	4	25.00
**Prior miscarriages**		
No	81	82.65
Yes	17	17.35
**Medical illness history**		
HIV	14	14.29
HPT	10	10.20
Diabetes	1	1.02
Epilepsy	1	1.02
**Substance use history**		
Alcohol	17	17.35
Tobacco (cigarettes)	16	16.33
Snuff (smokeless tobacco)	11	11.22
Cannabis	4	4.08

HPT, Hypertension.

Twenty-four participants had comorbid medical conditions, two of whom had two conditions (one with hypertension and HIV and the other with hypertension and diabetes). Thirty-seven participants reported using at least one substance during their pregnancy. Tobacco was the most used substance, either smoked or used in a dry form as ‘snuff’. We separated the two forms of use as there is less understanding regarding the harmful effects of snuff use in pregnancy. Of the participants who reported using more than one substance, tobacco and alcohol were the most frequently used combination.

### Trauma history

Fifty-three participants reported having either personally experienced or witnessed a traumatic event. In 14 women, the traumatic event was linked to previous obstetric or gynaecological complications or neonatal demise. Four women reported experiencing IPV and three had a history of childhood trauma (one of whom was currently exposed to IPV). One woman reported exposure to criminal violence and another to workplace-related conflict. The remaining participants reporting a history of trauma did not wish to disclose its nature.

### Post-traumatic stress disorder prevalence

The results of the PCL-5 questionnaire are presented in Table 3, including the total score and the number of participants who endorsed at least one item in each cluster (i.e. who rated their experience as moderately severe for one or more items in each cluster). A Cronbach’s alpha coefficient is a measure of internal consistency and a measure of scale reliability. A value of 0.97 indicates good internal consistency and reliability for the PCL-5 within our population sample.

**TABLE 3:** Post-traumatic stress disorder symptoms.

Variable	*N*	%	Mean	s.d
**Post-traumatic stress disorder (alpha = 0.97)**	-	-	13.8	18.1
PTSD negative	82	83.67	-	-
PTSD positive	16	16.33	-	-
**Cluster B intrusion**	-	-	3.58	4.68
No symptoms	61	62.24	-	-
Moderate	37	37.76	-	-
**Cluster C avoidance**	-	-	1.76	2.41
No symptoms	74	75.51	-	-
Moderate	24	24.49	-	-
**Cluster D cognition and mood changes**	-	-	5.12	7.40
No symptoms	62	63.27	-	-
Moderate	36	36.73	-	-
**Cluster E arousal and reactivity**	-	-	3.37	5.18
No symptoms	66	67.35	-	-
Moderate	32	32.65	-	-

PTSD, post-traumatic stress disorder; s.d., standard deviation.

The PCL-5 scores ranged from 0 to 71 with a mean score of 13.8 (s.d. = 18.1), and 16 of the 98 participants scored 31 or more. However, many women endorsed individual items, indicating the presence of PTSD symptoms even if the total score was less than 31. A score of 2 or more for an item (at least a moderate severity of the symptom) was reported by 37 participants for Cluster B (intrusion), 24 for Cluster C (avoidance), 36 for Cluster D (cognition and mood changes) and 32 for Cluster E (arousal and reactivity) symptoms.

Twelve participants met DSM-5 criteria for a diagnosis of PTSD, one of whom had a total score of only 26. Of the five participants with total scores of 31 or more who did not meet DSM-5 criteria, four had not endorsed two or more Cluster E (arousal and reactivity) symptoms and one did not endorse any Cluster B (intrusion) symptoms, which were all experienced but only mildly.

All four participants who had been exposed to IPV met DSM-5 criteria for PTSD, with total PCL-5 scores of 68, 61, 41, and 26. One participant with gynaecological trauma (a failed termination of pregnancy for the current pregnancy) met DSM-5 criteria for PTSD, with a PCL-5 score of 43, and one who had experienced a neonatal death secondary to eclampsia did not meet DSM-5 criteria, although she had a PCL-5 score of 35. None of the other women who provided details about the experienced traumatic event received a provisional diagnosis of PTSD on either scoring method. Of interest, only one of the eight women with prior contact with mental health services had PTSD (one of the women exposed to IPV who had previously received treatment for depression).

**FIGURE 1:**
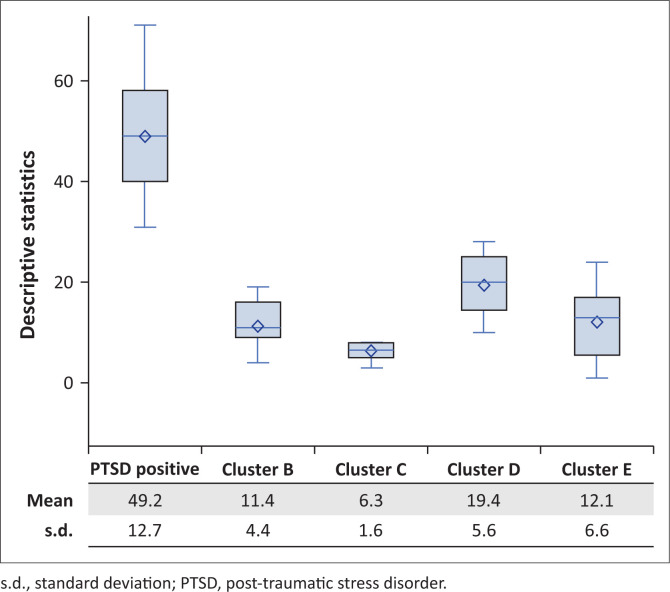
Mean and standard deviations of overall post-traumatic stress disorder and cluster scores among participants with post-traumatic stress disorder symptoms.

Among the 16 participants who screened positive for at least moderate PTSD when using the total score, the mean PCL-5 score was 49.2 (s.d. = 12.7), as illustrated in Figure 1. Of the clusters, Cluster D symptoms (cognition and mood changes) tended to be the most severe and Cluster C (avoidance) the least.

### Comparison between those with and those without post-traumatic stress disorder

No statistically significant differences were found between participants with and those without PTSD across sociodemographic, maternal health or substance use variables (Table 4). Although not significant, participants who were 18–24 years had a higher proportion of positive PTSD symptoms compared to negative PTSD symptoms (56.2% vs. 39.0%, *p* = 0.1097). All 16 participants who had positive PTSD symptoms were black people. A high proportion of participants who had positive PTSD symptoms attended post-high school (62.5%), had a monthly income of < R5999 (68.8%), did not receive a disability grant (62.5%) and were not currently employed (75.0%).

**TABLE 4:** Participants’ characteristics by post-traumatic stress disorder symptoms.

Variable	PTSD positive				PTSD negative				*p*-value
	*n*	%	Median	IQR	*n*	%	Median	IQR	
**Age (years)**	-	-	24	21.5–33.5	-	-	26	21–31	0.6577
18–24	9/16	56.25	-	-	32/82	39.02	-	-	0.1097
25–34	4/16	25.00	-	-	43/82	52.44	-	-	-
35–44	3/16	18.75	-	-	7/82	8.54	-	-	-
**Population group (%)**	-	-	-	-	-	-	-	-	-
Black people	16/16	100.0	-	-	66/82	80.49	-	-	0.2920
White people	0/16	0.00	-	-	10/82	12.20	-	-	-
Mixed race people	0/16	0.00	-	-	5/82	6.10	-	-	-
Asian people	0/16	0.00	-	-	1/82	1.22	-	-	-
**Relationship status (%)**	-	-	-	-	-	-	-	-	-
Single	7/16	43.75	-	-	27/82	32.93	-	-	0.2336
Living together	2/16	12.50	-	-	31/82	37.80	-	-	-
Married	7/16	43.75	-	-	23/82	28.05	-	-	-
Divorced	0/16	0.00	-	-	1/82	1.22	-	-	-
**Level of education (%)**	-	-	-	-	-	-	-	-	-
Mainstream Grade 0–7	0/16	0.00	-	-	2/82	2.44	-	-	0.1525
Mainstream Grade 8–11	0/16	0.00	-	-	14/82	17.07	-	-	-
Mainstream Grade 12	5/16	31.25	-	-	35/82	42.68	-	-	-
Special Needs Grade 10–12	1/16	6.25	-	-	4/82	4.88	-	-	-
Post High School	10/16	62.50	-	-	27/82	32.93	-	-	-
**Monthly income (%)**	-	-	-	-	-	-	-	-	-
< R5999	11/16	68.75	-	-	52/78	66.67	-	-	0.8685
R6000–R9999	4/16	25.00	-	-	23/78	29.49	-	-	-
≥ R10 000	1/16	6.25	-	-	3/78	3.85	-	-	-
**Housing type (%)**	-	-	-	-	-	-	-	-	-
Informal	0/16	0.00	-	-	4/82	4.88	-	-	0.6323
Rented room	6/16	37.50	-	-	39/82	47.56	-	-	-
Apartment/Flat	3/16	18.75	-	-	13/82	15.85	-	-	-
House	7/16	43.75	-	-	26/82	31.71	-	-	-
**Number of people in the household (%)**			2.50	1.50–4			3.00	2.00–4	0.8775
1	4/16	25.00	-	-	16/81	19.75	-	-	0.6498
2–4	9/16	56.25	-	-	55/81	67.90	-	-	-
5–9	3/16	18.75	-	-	10/81	12.35	-	-	-
**Do you receive a disability grant? (%)**	-	-	-	-	-	-	-	-	-
No	10/16	62.50	-	-	53/82	64.63	-	-	0.7855
Yes	0/16	0.00	-	-	2/82	2.44	-	-	-
Child care support grant	6/16	37.50	-	-	27/82	32.93	-	-	-
**Currently employed? (%)**	-	-	-	-	-	-	-	-	-
No	12/16	75.00	-	-	54/82	65.85	-	-	0.4754
Yes	4/16	25.00	-	-	28/82	34.15	-	-	-
**Gestational age (months)**	-	-	-	-	-	-	-	-	-
Unsure	2/16	12.50	-	-	16/82	19.51	-	-	0.6396
3	1/16	6.25	-	-	12/82	14.63	-	-	-
3–6	6/16	37.50	-	-	28/82	34.15	-	-	-
6–9	7/16	43.75	-	-	26/82	31.71	-	-	-
**Number of other children**	-	-	-	-	-	-	-	-	-
None	8/16	50.00	-	-	41/82	50.00	-	-	0.9967
One	5/16	31.25	-	-	25/82	30.49	-	-	-
More than one	3/16	18.75	-	-	16/82	19.51	-	-	-
**Prior pregnancy complications**	-	-	-	-	-	-	-	-	-
No	13/16	81.25	-	-	69/82	84.15	-	-	0.7743
Yes	3/16	18.75	-	-	13/82	15.85	-	-	-
**Prior miscarriages**	-	-	-	-	-	-	-	-	-
No	13/16	81.25	-	-	68/82	82.93	-	-	0.8713
Yes	3/16	18.75	-	-	14/82	17.07	-	-	-
**Medical illness history**	-	-	-	-	-	-	-	-	-
HIV	4/16	25.00	-	-	10/82	12.20	-	-	0.1806
HPT	1/16	6.25	-	-	9/82	10.98	-	-	0.9999
Diabetes	0/16	0.00	-	-	1/82	1.22	-	-	-
Epilepsy	0/16	0.00	-	-	1/82	1.22	-	-	-
**Substance use history**	-	-	-	-	-	-	-	-	-
Alcohol	5/16	31.25	-	-	12/82	14.63	-	-	0.1454
Tobacco (cigarettes)	3/16	18.75	-	-	13/82	15.85	-	-	0.7216
Snuff (smokeless tobacco)	0/16	0.00	-	-	11/82	13.41	-	-	-
Cannabis	1/16	6.25	-	-	3/82	3.66	-	-	0.5158

PTSD, Post-traumatic stress disorder; IQR, inter-quartile range; HPT, hypertension.

While there were no differences between the two groups in terms of each substance used, drinking two units of alcohol per day was significantly more common among those with PTSD compared to those without (25.0% vs. 4.9%, *p* = 0.0072) (Table 5).

**TABLE 5:** Quantity of substance use per day by post-traumatic stress disorder symptoms.

Variable	Overall		PTSD positive		PTSD negative		*p*-value
	*N*	%	*n*	%	*n*	%	
**Alcohol (%)**							
1 unit a day	1/98	1.02	0/16	0.00	1/82	1.22	-
2 units a day	8/98	8.16	4/16	25.00	4/82	4.88	**0.0072**
3 units a day	4/98	4.08	1/16	6.25	3/82	3.66	0.6318
4 units a day	1/98	1.02	0/16	0.00	1/82	1.22	-
Unwilling to quantify	3/98	3.06	0/16	0.00	3/82	3.66	-
Not applicable	81/98	82.65	11/16	68.75	70/82	85.37	0.1084
**Tobacco (cigarettes) (%)**							
< 10 units a day	6/98	6.12	0/16	0.00	6/82	7.32	0.2207
10–19 units a day	3/98	3.06	1/16	6.25	2/82	2.44	-
20 units a day	3/98	3.06	0/16	0.00	3/82	3.66	-
Not specified	4/98	4.08	2/16	12.50	2/82	2.44	-
Not applicable	82/98	83.67	13/16	81.25	69/82	84.15	-
**Snuff (smokeless tobacco) (%)**							
Hourly	5/98	5.10	0/16	0.00	5/82	6.10	0.6594
Every 2 h	1/98	1.02	0/16	0.00	1/82	1.22	-
Every 4 h	2/98	2.04	0/16	0.00	2/82	2.44	-
Not specified	3/98	3.06	0/16	0.00	3/82	3.66	-
Not applicable	87/98	88.78	16/16	100.0	71/82	86.59	-
**Cannabis (%)**							
1 joint a day	1/98	1.02	0/16	0.00	1/82	1.22	0.6572
Unwilling to quantify	3/98	3.06	1/16	6.25	2/82	2.44	-
Not applicable	94/98	95.92	15/16	93.75	79/82	96.34	-

Note: Bold values indicate the significance of 2 units of alcohol in participants expressing positive PTSD symptoms - our one significant finding.

PTSD, post-traumatic stress disorder.

### Factors associated with post-traumatic stress disorder

Multivariate analysis revealed alcohol use as the only factor significantly associated with having at least moderate PTSD (Relative Risk 2.7, 95% CI 1.1–7.0, *p* = 0.0364) (Table 6).

**TABLE 6:** Risk factors associated with post-traumatic stress disorder.

Variable	Univariate			Multivariate		
	RR	95% CI	*p*-value	RR	95% CI	*p*-value
**Age (in years)**						
25–34 vs. 18–24	0.3877	0.1290–1.1657	0.0916	0.3608	0.1200–1.0848	0.0695
35–44 vs. 18–24	1.3667	0.4509–4.1421	0.5808	1.6397	0.4456–6.0334	0.4569
**Relationship status**						
Living together vs. Single	0.2944	0.0659–1.3149	0.1093	-	-	-
Married vs. Single	1.1333	0.4492–2.8595	0.7910	-	-	-
**Monthly income**						
R6000–R9999 vs. R5999	0.8485	0.2964–2.4291	0.7595	-	-	-
≥ R10 000 vs. < R5999	1.4318	0.2414–8.4927	0.6927	-	-	-
**Currently employed?**						
No vs Yes	1.4545	0.5091–4.1561	0.4842	-	-	-
**Gestation age**						
3 months vs. Unsure	0.6923	0.0700–6.8502	0.7532	-	-	-
3–6 months vs. Unsure	1.5882	0.3562–7.0814	0.5441	-	-	-
6–9 months vs. Unsure	1.9091	0.4421–8.2432	0.3863	-	-	-
**Prior pregnancy complications**						
Yes vs. No	1.1827	0.3800–3.6809	0.7721	1.2301	0.3591–4.2138	0.7416
**Prior Miscarriages**						
Yes vs. No	1.0995	0.3512–3.4425	0.8705	-	-	-
**Medical illness history**						
HPT	0.5867	0.0864–3.9843	0.5853	-	-	-
HIV	2.0000	0.7506–5.3288	0.1657	-	-	-
**Substance use history**						
Alcohol	2.1658	0.8642–5.4278	0.0992	**2.7367**	**1.0660–7.0261**	**0.0364**
Tobacco (cigarettes)	1.1827	0.3800–3.6809	0.7721	-	-	-
Cannabis	1.5667	0.2696–9.1028	0.6170	-	-	-

HPT, hypertension; RR, relative risk; CI, confidence interval.

### Distress protocol intervention

A total of 53 (54%) participants exhibited some distress during or after the interview and were referred to a mental health professional. In addition to those referred for management of their PTSD symptoms, others were referred for assistance with poor social support, family stressors and persistent substance use. While several referrals were to a registered counsellor or social worker, 30 were referred to the specialist mental health services.

## Discussion

In this questionnaire-based study among pregnant women attending a community-based antenatal clinic in South Africa, PTSD was found to be highly prevalent at 16% of the participants. Alcohol use in pregnancy was the only risk factor significantly associated with PTSD in our study sample. In general, our sample is reflective of the broader population in Gauteng province, being mostly black African but with a slightly higher mixed race population than the rest of the province.^16^ Although 38% of our sample reported some tertiary education, unemployment was double the national official unemployment rate of 33%.^18^

Our prevalence rate of 16% is closer to the 19% prevalence found among high-risk antenatal populations by Yildiz et al., compared to the 3.3% they found among community-based or general maternity populations.^13^ This is very disturbing, considering that our population was community-based and may be a reflection of the high levels of violence in South Africa.

Psychological trauma, PTSD and associated risk factors among pregnant women in the Western Cape province of South Africa were investigated in the Drakenstein Child Health Study.^19^ In this study, lifetime trauma exposure was reported in approximately two-thirds (67%) of the sample, with a 19% prevalence of lifetime or recurrent PTSD. They found that women with PTSD were more likely to have depression, suicidal thoughts or behaviour, and/or alcohol dependence than those without PTSD. Risk factors for PTSD among the women with a history of trauma exposure were childhood trauma and recent stressful events. The finding of childhood trauma as a risk factor in the Drakenstein Child Health Study is consistent with the meta-analysis conducted by Yildiz et al. As most of our trauma-exposed participants did not wish to disclose the nature of the trauma, we were unable to evaluate childhood trauma as a risk factor.

As with the Drakenstein Child Health Study, we found a significant risk of alcohol abuse among those with PTSD. A positive relationship between alcohol use and PTSD has been well documented internationally in general population surveys^20^ and among people with PTSD or with an alcohol use disorder.^21^ However, directional causality is unclear. Although alcohol may be used as a form of self-medication for PTSD symptoms, using alcohol may increase the risk of exposure to traumatic events and therefore PTSD. Overall, 17% of our sample population used alcohol while pregnant. This is much higher than an estimated global rate of alcohol use during pregnancy of 9.8%^22^ and the national rate of 3.7% found in the population-based South African National Health and Nutrition Examination Survey (SANHANES) conducted between 2011 and 2012.^23^ Of note, the SANHANES study found alcohol use in pregnancy to be associated with being of the mixed race South African population group, unemployed, and/ or having PTSD, all of which were prevalent in our study sample.

Of serious concern is the poor identification and referral of mental health conditions in routine antenatal care, with only six participants having scored at least one on the routine mental health screen. In addition, only eight participants reported any previous contact with mental health services. The lack of mental health care is consistent with the treatment gap of 75% found in the SASH study for common mental disorders.^3^ The need to address common and severe mental health conditions as well as substance use during pregnancy has been emphasised in the South African Maternal, Neonatal and Child Health Policy.^24^ Detailed implementation guidance has also been provided in a clinical programme guideline.^25^ However, important barriers to such implementation remain, including inadequate training and support of antenatal staff, overburdened clinics, and high levels of stigma around mental health conditions.^26^ Finally, access to mental health care practitioners at the district level is inadequate, including in Gauteng province, which has actively promoted integrated primary mental health care and has a limited community psychiatric service.^27^

### Study limitations

This study has several limitations. The design of the study only allowed for general estimates of PTSD prevalence; thus, a temporal relationship or causality may not be inferred from the results. Another limitation was the presence of selection bias which meant that those participants who could not participate in the study were not screened and therefore may be at risk of PTSD and its associated complications. Similarly, only women who attended the clinic during data collection for their antenatal check-up were screened, leaving out those women who either did not visit the clinic or those who do not attend any antenatal clinic. Finally, the sample population was drawn from a peri-urban district in the most industrialised province of South Africa, and therefore, the findings may not be generalisable to the rest of the country.

## Conclusion

Post-traumatic stress disorder is highly prevalent among pregnant women attending a community-based antenatal clinic in South Africa and is significantly associated with alcohol use during pregnancy. However, identification of mental health conditions and referral for appropriate interventions by antenatal clinic staff are poor, notwithstanding an emphasis on maternal mental health care in national policy and guidelines.

### Recommendations

Further research is needed to explore means of strengthening maternal mental health care. However, increasing the capacity of antenatal nursing staff through additional training and improved human resources is likely to help. In particular, the addition of registered counsellors and/or social workers to antenatal clinics and midwifery units would be beneficial.
